# Machine learning-based radiomics to differentiate immune-mediated necrotizing myopathy from limb-girdle muscular dystrophy R2 using MRI

**DOI:** 10.3389/fneur.2023.1251025

**Published:** 2023-10-23

**Authors:** Ping Wei, Huahua Zhong, Qian Xie, Jin Li, Sushan Luo, Xueni Guan, Zonghui Liang, Dongyue Yue

**Affiliations:** ^1^Department of Radiology, Jing’an District Center Hospital of Shanghai, Fudan University, Shanghai, China; ^2^Department of Neurology, Huashan Hospital, Fudan University, Shanghai, China; ^3^Department of Neurology, Jing’an District Center Hospital of Shanghai, Fudan University, Shanghai, China

**Keywords:** immune-mediated necrotizing myopathy, limb-girdle muscular dystrophy, radiomics, muscle MRI, machine learning, fatty infiltration

## Abstract

**Objectives:**

This study aimed to assess the feasibility of a machine learning-based radiomics tools to discriminate between Limb-girdle muscular dystrophy R2 (LGMDR2) and immune-mediated necrotizing myopathy (IMNM) using lower-limb muscle magnetic resonance imaging (MRI) examination.

**Methods:**

After institutional review board approval, 30 patients with genetically proven LGMDR2 (12 females; age, 34.0 ± 11.3) and 45 patients with IMNM (28 females; age, 49.2 ± 16.6) who underwent lower-limb MRI examination including T1-weighted and interactive decomposition water and fat with echos asymmetric and least-squares estimation (IDEAL) sequences between July 2014 and August 2022 were included. Radiomics features of muscles were obtained, and four machine learning algorithms were conducted to select the optimal radiomics classifier for differential diagnosis. This selected algorithm was performed to construct the T1-weighted (TM), water-only (WM), or the combined model (CM) for calf-only, thigh-only, or the calf and thigh MR images, respectively. And their diagnostic performance was studied using area under the curve (AUC) and compared to the semi-quantitative model constructed by the modified Mercuri scale of calf and thigh muscles scored by two radiologists specialized in musculoskeletal imaging.

**Results:**

The logistic regression (LR) model was the optimal radiomics model. The performance of the WM and CM for thigh-only images (AUC 0.893, 0.913) was better than those for calf-only images (AUC 0.846, 0.880) except the TM. For “calf + thigh” images, the TM, WM, and CM models always performed best (AUC 0.953, 0.907, 0.953) with excellent accuracy (92.0, 84.0, 88.0%). The AUCs of the Mercuri model of the calf, thigh, and “calf + thigh” images were 0.847, 0.900, and 0.953 with accuracy (84.0, 84.0, 88.0%).

**Conclusion:**

Machine learning-based radiomics models can differentiate LGMDR2 from IMNM, performing better than visual assessment. The model built by combining calf and thigh images presents excellent diagnostic efficiency.

## 1. Introduction

Limb-girdle muscular dystrophy R2 (LGMDR2) (previously called LGMD2B) is genetically determined muscular dystrophy with autosomal recessive transmission due to mutations in the DYSF gene leading to sarcolemma repair abnormalities and secondary inflammatory activation (1). Its clinical features predominantly include progressive weakness and atrophy of proximal limb muscles (2). Immune-mediated necrotizing myopathy (IMNM) is a lately identified subtype of idiopathic inflammatory myopathies (IIMs), which are a group of autoimmune diseases usually characterized by auto-antibodies including 3-hydroxy-3-methylglutaryl-coenzyme A reductase (HMGCR) or the signal recognition particle (SRP) (3). Moreover, its primary clinical manifestation is rapidly progressive muscle weakness (3). In recent years, many researchers noticed that LGMDR2 most frequently mimics IMNM because they both have onset in adulthood and have similar clinicopathological features, including progressive proximal muscle weakness, elevated creatine kinase (CK) and scattered myonecrosis or inflammatory infiltrates on a muscle biopsy, which leads quickly to clinical misdiagnosis (4, 5). Nevertheless, they differ in their management. Patients with LGMDR2 depend on supportive care only, while IMNM patients respond to early aggressive immunotherapies (3, 6). Thus, timely and accurate discrimination between two diseases is essential for predicting clinical courses and making treatment decisions.

Muscle MRI is capable of clearly demonstrating muscle edema, atrophy or fat replacement, becoming an indispensable tool for diagnosing myopathies, and therefore it contributes to researching the particular pattern of muscle involvement in various muscle diseases (7–9). Although previous studies have compared characteristic muscle MRI performance in LGMDs-and IIM-patients (5, 10, 11), the similarity and complexity of these patterns pose great challenges for radiological diagnosis. Mercuri et al. (12) published the Mercuri scale that has been frequently used to grade intramuscular fatty infiltration based on conventional T1-weighted sequence, but the method depends on the human visual system, which is subjective and does not enable quantification. Thus, the quantitative assessments with MRI need to be considered in the early diagnosis and further monitoring of myopathies.

Radiomics is a novel and attractive data analysis technique that can mine a mass of high-throughput quantitative image features from standard medical imaging, reflecting subtle pathophysiologic features often imperceptible to humans (13, 14). With an increasing incidence of muscle diseases and a further understanding of the relationship between muscle imaging and pathophysiology currently, attention has gradually been paid to radiomics analysis of musculoskeletal imaging (15). To date, a few studies have applied radiomics assessments to myopathy, which has shown promising results regarding the ability of this technique to help in the differentiation between neurogenic and myogenic diseases using muscle ultrasound (16), or in the diagnosis of IIM (11) and inherited myopathy using muscle MRI (16).

However, studies are yet to differentiate between LGMDR2 and IMNM by exploiting radiomics analysis. We hypothesize that the MRI-based radiomics tool could discriminate LGMDR2 from IMNM. The purpose of this study was to establish machine learning-based MRI radiomics models and evaluate the capability of these to distinguish between LGMDR2 and IMNM, as well as compare with the Mercuri model based on the assessment of radiologists.

## 2. Materials and methods

### 2.1. Patients

**Figure 1:**
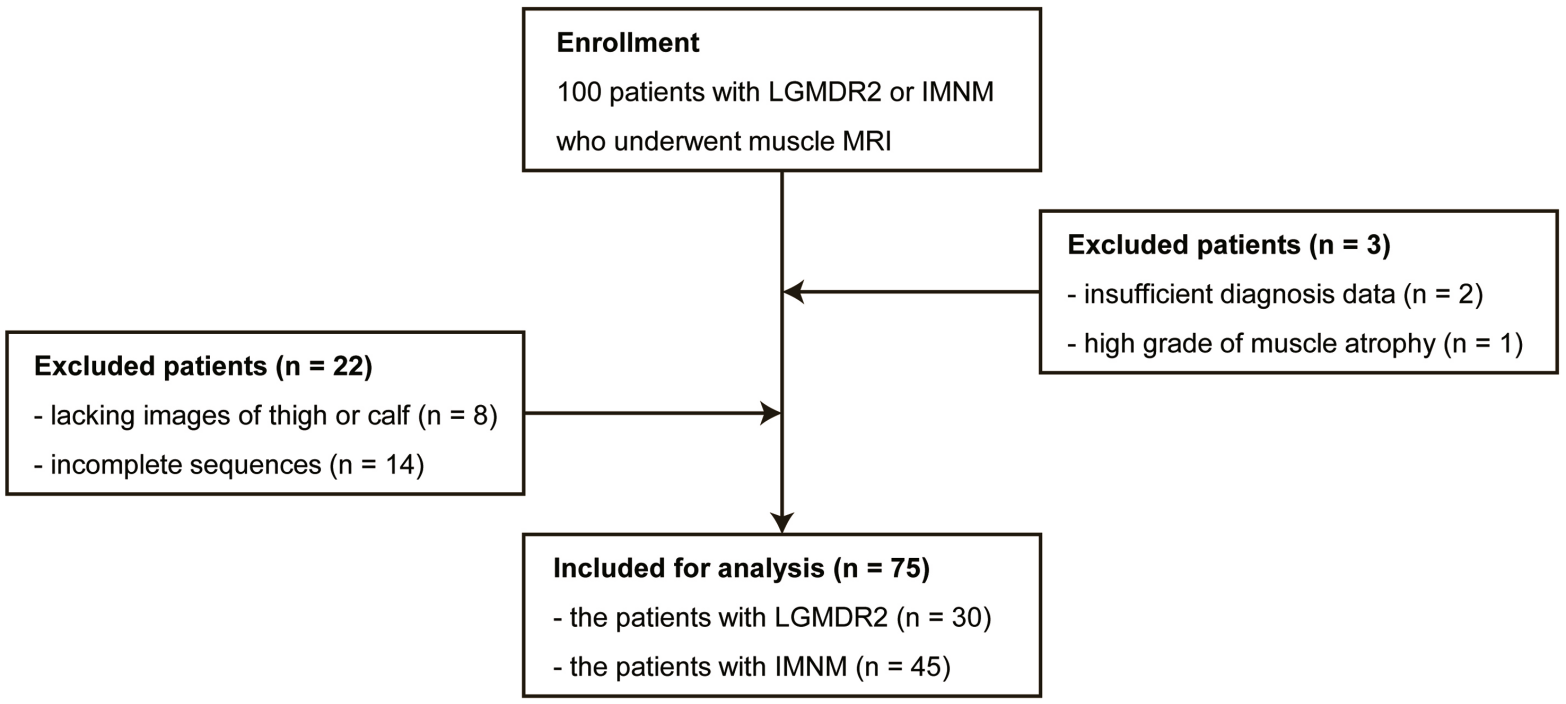
The flowchart shows the enrollment of LGMDR2 and IMNM patients.

This retrospective study was approved by the Institutional Review Board of Huashan Hospital, Fudan University (KY2019-409), and exempt from informed consent. Data from LGMDR2 or IMNM patients who underwent lower-limb muscle MRI in our Radiology Department between July 2014 and August 2022 was retrieved. Diagnoses of LGMDR2 were determined based on clinical evaluation and genetic testing confirmed by pure or compound heterozygous mutation in the DYSF gene. For diagnoses of IMNM, results of clinical judgment, antibody testing, and muscle biopsy were used, as suggested by the European Neuromuscular Centre International Workshop diagnostic criteria (17). Patients who underwent calf and thigh MR examination including T1-weighted sequence and T2-weighted IDEAL sequence, were enrolled. The main exclusion criteria were as follows: (I) insufficient diagnosis data; (II) high grade of muscle atrophy (difficulty in segmentation); (III) lacking images of thigh or calf; (IV) incomplete sequences. As illustrated in Figure 1, 30 patients were included in the LGMDR2 group, and 45 patients were enrolled in the IMNM group after careful selection.

### 2.2. MRI acquisitions

All MRI examinations were performed by a 3.0-Tesla scanner (Discovery MR750; GE Healthcare, Chicago, Illinois) equipped with a GE 8-channel body coil. The lower-limb muscle imaging protocol included an axial T1-weighted FSE sequence (flip angle: 111°; repetition time/echo time: 676/14 ms; acquisition matrix: 320 × 224; field of view [FOV]: 380 × 228 mm; slice thickness/spacing: 6/18 mm) and an axial T2-weighted FSE-IDEAL sequence (flip angle: 111°; repetition time/echo time: 2545/85 ms; acquisition matrix: 320 × 256; FOV: 380 × 228 mm; slice thickness/spacing: 6/18 mm). The thigh images were obtained at levels from the lesser trochanter through the femoral condyles, and the calf images were obtained at levels from the tibiofibular joint to the soleus myotendinous junction. FSE-IDEAL sequence, an innovative 3-point Dixon technique, effectively separates the fat signal from the water signal to reconstruct water-only and fat-only images (18).

### 2.3. Image assessment and segmentation

**Figure 2:**
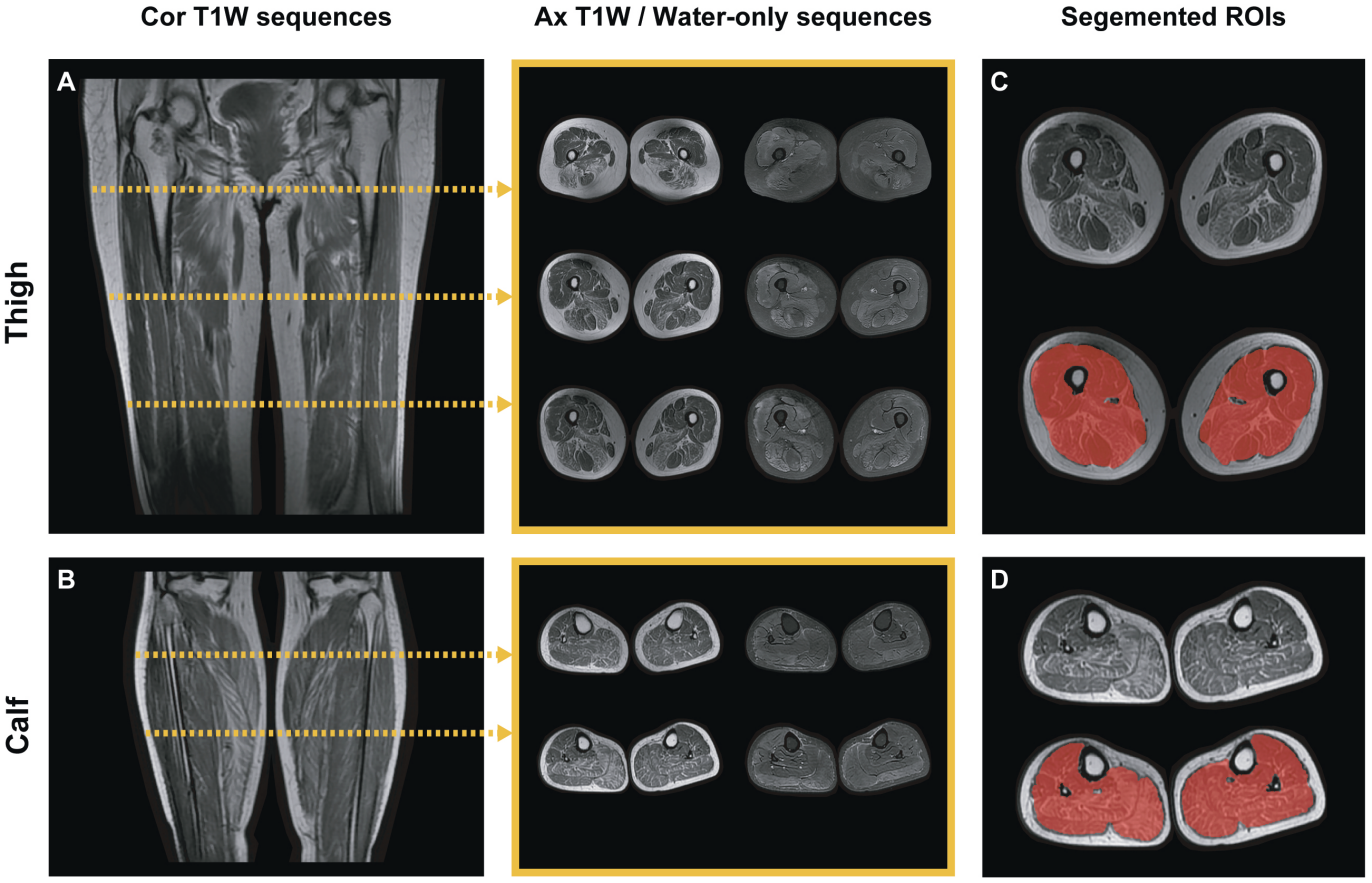
Lower-limb muscle MR image selection and segmentation. **(A,B)** Two images showed the coronal T1-weighted calf and thigh images. For each patient, five slices in different areas were selected for radiomics analysis: three slices of thighs and two slices of calves for each sequence (T1-weighted and water-only). The middle showed the selected slices of axial T1-weighted and water-only images, respectively. **(C,D)** The global area of the muscles in each selected slice was segmented as calf or thigh ROI (red-shaded area), excluding the epimysium.

Two radiologists (Reader 1 and 2) with over 10 years of experience in musculoskeletal MRI participated in slice selection and segmentation using ITK-SNAP (version 3.8.0; www.itksnap.org) software. Muscle MR images from each patient have been imported into the software, and then the water-only images were automatically aligned to T1-weighted images. For each subject, three slices were selected from the proximal, middle, and distal bilateral thighs, respectively, and two from the proximal and middle of the bilateral calves. This selection ensured that a two-dimensional region of interest (ROI) would involve the following muscles: thigh: rectus femoris (RFEM), vastus medialis (VME), vastus intermedius (VIN), vastus lateralis (VLA), sartorius (SAR), gracilis (GR), adductor longus (AL), adductor magnus (AM), semimembranosus (SMB), semitendinosus (SMT), biceps femoris (HBF); and calf: tibialis anterior (TA), peroneal muscle (PER), tibialis posterior (TP), soleus (SOL), gastrocnemius (medial head [GASM] and lateral head [GASL]). With the water-only images as references, they individually and manually segmented the ROI on the T1-weighted images of every patient that covered the global muscle area of each selected slice and excluded the epimysium (Figure 2). Reyngoudt et al. reported that the MRI fat fraction analysis with global muscle segment in the calf or thigh has shown the capability to be nearly as sensitive as individual muscle ROIs in most muscular diseases, which is an easier and faster segmentation method (19). Subsequently, the delineated ROIs based on the T1-weighted images were copied to the same location of the water-only images. And minor revision was performed to the ROIs to erase unnecessary regions, and add missed muscle areas on the water-only images. The ROIs of two regions, including thigh and calf ROIs, were then obtained. Reader 1 performed muscle segmentation on the same image slices again with a minimum interval of 2 months. The intraclass correlation coefficient (ICC) were calculated to evaluate the reliability and repeatability of features by different segmentation.

The two radiologists individually evaluated the T1-weighted images of the above-mentioned muscles and graded muscular fatty infiltration based on the modified Mercuri scale score (12, 20, 21) as follows: score 0, normal appearance; score 1, punctate T1 hyperintense lesion; score 2, scattered T1 hyperintense lesion (<30% muscle bulk); score 3, small confluent lesion of T1 hyperintensity (30–60% muscle bulk); score 4, large confluent lesion of T1 hyperintensity (>60% muscle bulk) and score 5, diffuse T1 hyperintense in the global muscle. Both radiologists were blinded to the clinical information (e.g., name, age, sex, clinical diagnosis), and evaluations were performed at the same levels where the ROIs were segmented on the T1-weighted images.

### 2.4. Radiomics feature extraction

Before the feature extraction, all MR images were resampled to the same resolution (3 × 3 × 3 mm) with linear interpolation to avoid data heterogeneity bias. Radiomics features were extracted from the thigh and calf ROIs of the T1-weighted and water-only images, respectively, using the “PyRadiomics” package in Python 3.9.13 (www.python.org). For each patient, 2,024 radiomics calf or thigh features were extracted from calf-only or thigh-only ROIs (each ROI including 1,012 T1-weighted and 1,012 water-only features, respectively). An early fusion was used to combine T1-weighted or water-only features of calf and thigh images into “calf + thigh” combined features (22). The extracted features were divided into three sets (23, 24): (1) First-order features describe the distribution of voxel intensities. (2) Texture features represent the second and high-order spatial heterogeneity of the intensity level containing gray level dependence matrix (GLDM), gray level co-occurrence matrix (GLCM), gray level run length matrix (GLRLM), gray level size zone matrix (GLSZM), and neighborhood gray-tone difference matrix (NGTDM). (3) And transform features from three filters including local binary pattern (LBP), gradient and wavelet. The gradient filter was to replace all voxel values in an area with the local gradient value of the image (e.g., maximum-minimum value). And the LBP features represent a comparison of central pixels and their surrounding pixels. For wavelet transforms, each image was transformed in the x, y, and z directions using a low or high band pass filter.

### 2.5. Feature selection

**Figure 3:**
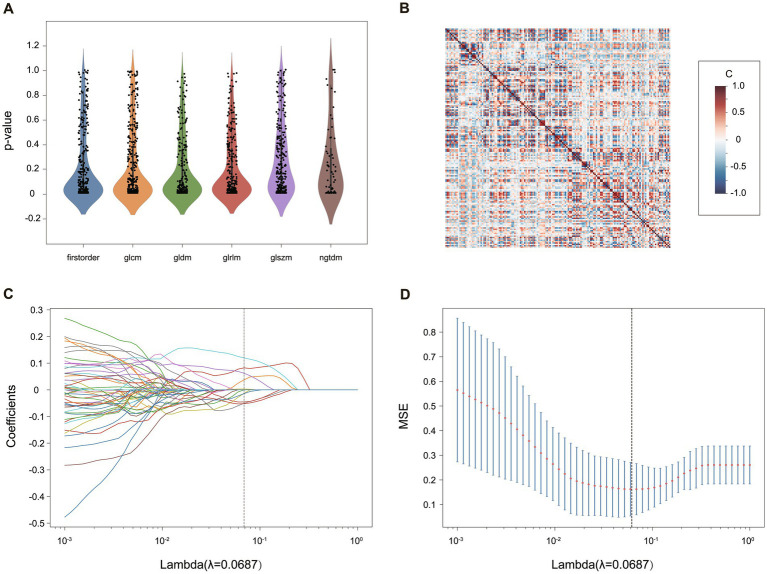
The process of radiomics feature selection in the “calf + thigh” images for the TM. **(A)** The statistical distribution of the extracted radiomics features. The *p*-value > 0.05 of radiomics features was removed. **(B)** The heatmap of radiomics features after correlation filtering. The correlation coefficient [C] ranges between −1 and +1. The higher the C, the more redundant the feature was. **(C)** The coefficient profile plot and **(D)** the 10-fold cross-validation plot of the LASSO regression model. The vertical lines on **(C,D)** determine the optimal *λ*.

After all the features were normalized by the Z score, the selection was performed in the following four steps, illustrated in Figure 3. Firstly, only features with ICC ≥ 0.81 (excellent stability) were retained. Next, Mann Whitney *U*-test was conducted to choose the significant features with a *p*-value less than 0.05 (Figure 3A). Then, for features with high repeatability, Spearman’s rank correlation coefficient was also used to calculate the correlation between features (Figure 3B), and only one of the features with a correlation coefficient greater than 0.9 between any two features is retained (25). Finally, the most minor absolute shrinkage and selection operator (LASSO) regression model with 10-fold cross-validation was used to select the remaining features. Depending on the regulation weight *λ*, LASSO shrinks all regression coefficients toward zero and precisely sets the coefficients of many irrelevant features to zero (26) (Figures 3C,D). The retained features with non-zero coefficients were retained for regression model fitting. Subsequently, we obtained the most representative features for each patient which were used to establish classification models (Table 1).

**Table 1:** The selected features of different ROIs.

ROIs		Selceted features	
Calf	T1W (*n* = 16)	gradient_glszm_SmallAreaLowGrayLevelEmphasis	lbp_2D_firstorder_Kurtosis
		lbp_2D_firstorder_InterquartileRange	lbp_2D_gldm_DependenceVariance
		lbp_2D glrlm_LowGraylevelRunEmphasis	wavelet_LHL_firstorder_Median
		wavelet_LHL_firstorder_Mean	wavelet_LHL_glcm_Imc2
		wavelet_LLH_glszm_ZoneEntropy	wavelet_LLL_ngtdm_Complexity
		wavelet_LLL_glszm_ZoneVariance	wavelet_HHL_glszm_ZoneEntropy
		wavelet_LHH_glrlm_GrayLevelVariance	wavelet_HLL_ngtdm_Contrast
		wavelet_HHL_glrlm_LongRunHighGrayLevelEmphasis	wavelet_HHL_firstorder_Mean
	Water-only (*n* = 9)	gradient_glszm_SizeZoneNonUniformity	wavelet_LHH_firstorder_Mean
		wavelet_LLH_glcm_InverseVariance	wavelet_LHL_firstorder_Mean
		wavelet_LLL_firstorder_10Percentile	wavelet_LLL_ngtdm_Contrast
		wavelet_LLL_glszm_LargeAreaHighGrayLevelEmphasis	wavelet_LLL_glcm_Idn
		wavelet_HLH_gldm_LargeDependenceEmphasis	
Thigh	T1W (*n* = 8)	gradient_glcm_Imc2	gradient_ngtdm_Strength
		wavelet_LLH_glszm_GrayLevelVariance	wavelet_LLH_glrlm_RunEntropy
		wavelet_LLL_firstorder_Kurtosis	wavelet_HLL_glcm_ClusterShade
		wavelet_HLL_glszm_ZoneEntropy	wavelet_HHL_firstorder_Mean
	Water-only (*n* = 14)	gradient_glszm_SizeZoneNonUniformity	wavelet_LHL_glcm_Idmn
		wavelet_LHH_glcm_MaximumProbability	wavelet_LHH_glcm_ClusterShade
		wavelet_LHH_glszm_GrayLevelNonUniformity	wavelet_HLL_firstorder_Maximum
		wavelet_LLH_glszm_SizeZoneNonUniformity	wavelet_HLH_ngtdm_Complexity
		wavelet_LLL_glszm_HighGrayLevelZoneEmphasis	wavelet_HHL_firstorder_Median
		wavelet_LLL_glszm_LargeAreaHighGrayLevelEmphasis	wavelet_HHL_firstorder_Skewness
		wavelet_LLL_glszm_SizeZoneNonUniformity	wavelet_HHL_glcm_ClusterShade

T1W, T1-weighted. glszm, gray level size zone matrix; lbp, local binary pattern; gldm, gray level dependence matrix; glrlm, gray level run length matrix; glcm, gray level co-occurrence matrix; Imc, Informational measure of correlation; ngtdm, neighborhood gray-tone difference matrix; Idn, Inverse difference normalized; Idmn, Inverse difference moment normalized.

### 2.6. Model building and evaluation

All patients are divided into a training and a test cohort by a 7:3 ratio. Different radiomics models were developed and tested, respectively, to differentiate LGMDR2 and IMNM based on the following four machine learning classification algorithms: LR, k-Nearest Neighbors (kNN), random forest (RF), and eXtreme Gradient Boosting (XGBoost). The radiomics T1-weighted sequence model (TM), the water-only sequence model (WM), and the “combined” sequence model (the fusion of T1-weighted and water-only sequences, CM) were built with calf, thigh and “calf + thigh” muscular images separately based on different selected features. The Mercuri diagnosis model for differentiating LGMDR2 and IMNM were built with the four machine learning classifiers by the Mercuri score in each muscle of the calf-only, thigh-only and “calf + thigh” images, respectively. Their respective area under the receiver operating characteristic (ROC) curve (AUC) values and accuracy (ACC) were used to evaluate the performance of each model. And the machine learning classifier with the highest AUC of the test cohort was used to construct the optimal prediction models. The AUC, accuracy, sensitivity, specificity, and F1 measure were used to evaluate the classification performance and goodness of fit of the various models. All model building was developed in Python.^1^

### 2.7. Statistical analysis

All statistical analysis were performed using SPSS (version 23.0; SPSS Inc., Chicago, IL, United States). Differences in fatty infiltration scores for each muscle of thighs and calves between LGMDR2 and IMNM based on the Mercuri scale scores were assessed in terms of the Mann Whitney *U*-test. A statistically significant difference was considered at *p* < 0.05. ICC was used to evaluate intra-observer and inter-observer agreements for radiomics features obtained from different segmentation, interpreted as follows: 1.0, perfect; 0.81–0.99, almost perfect; 0.61–0.80, substantial; 0.41–0.60, moderate; 0.21–0.40, fair; and 0.20 or less, slight agreement (27). Inter-observer agreement of Mercuri scale assessment was performed using Cohen’s kappa analysis, and the kappa value >0.80 was considered to represent almost perfect agreement (28).

## 3. Result

### 3.1. Patient characteristics

Thirty patients with genetically proven LGMDR2 and 45 with IMNM (15 with the anti-SRP autoantibody, 15 with the anti-HMGCR autoantibody, and 15 with seronegative IMNM) were selected from one hundred patients who underwent muscle MRI examinations for myopathy between July 2014 and August 2022. Patients with LGMDR2 were younger than those with IMNM (*p* < 0.024; LGMDR2 patients: median ± standard deviation, 34.03 ± 11.266; IMNM patients: median ± standard deviation; 49.20 ± 16.583). The IMNM patients showed a greater female prevalence than the LGMDR2 patients, while there was no significant difference between the two diseases (*p* < 0.059). LGMDR2 had the most apparent fat infiltration in the posterior muscle group of the thigh and calf. In IMNM, the posterior group of thigh muscle was also more heavily affected by fat than any other muscle group. Most of the muscles in the thighs and calves except sartorius, gracilis and tibialis posterior showed higher scores of fat substitution in LGMDR2 than IMNM (*p* < 0.05).

### 3.2. Intra-observer and Inter-observer agreement evaluation

Mercuri scores of each muscle graded separately by two radiologists showed almost perfect agreement, and the Kappa value ranged from 0.826 to 0.933. Features derived from the ROIs segmented separately by two radiologists showed almost perfect agreement, and the mean ICC values in the intra-observer agreement ranged from 0.895 to 0.917 and from 0.833 to 0.873 in the inter-observer reproducibility test (Table 2). Thus, all statistical analyses were based on the results of the first feature extraction segmented by reader 1. For calf-only ROIs, 834 T1-weighted and 874 water-only features had excellent intra-reader and inter-reader reproducibilities (ICC ≥ 0.81), respectively, with 770 T1-weighted and 800 water-only features for thigh-only ROIs.

**Table 2:** Intra-observer and inter-observer of muscular segmentation.

Images		Intra-observative agreement		Inter-observative agreement	
		ICC	95%CI	ICC	95%CI
Calf	T1-weighted	0.917	0.902–0.932	0.867	0.851–0.881
	Water-only	0.912	0.897–0.928	0.873	0.857–0.889
Thigh	T1-weighted	0.895	0.880–0.909	0.835	0.816–0.854
	Water-only	0.897	0.882–0.912	0.833	0.813–0.852

ICC, intraclass correlation coefficient; CI, confidence intervals.

### 3.3. Radiomics feature selection and optimal machine learning algorithm

In this study, radiomics features were extracted from the different ROIs. After excluding features with ICC < 0.81, *p*-value > 0.05 in *U*-test statistical test (Figure 3A) and Pearson correlation coefficients >0.9, a heatmap of selected features depicts little redundancy (Figure 3B). All features with non-zero coefficients were chosen for model building after the LASSO regression analysis, and then 16 T1-weighted and 9 water-only features were finally retained for calf-only ROIs, 8 and 14 for thigh-only ROIs, respectively (Table 1). The AUC and accuracy of the radiomics TM, WM, CM and the Mercuri model constructed by four machine learning algorithms are listed in Table 3. The LR algorithm performed the highest AUC (0.953) and accuracy (92.0%) in the TM for “calf + thigh” images. Therefore, LR was regarded as the optimal machine-learning algorithm for subsequent model analysis. Also, the LR classifier is the best option for Mercuri model. Figure 3 shows the feature selection process in the TM for “calf + thigh” images. And the positive coefficients features contribute most to the best model after LASSO analysis.

**Table 3:** The performances of various machine learning algorithms.

Models		LR		kNN		RF		XGBoost	
		AUC	ACC (%)	AUC	ACC (%)	AUC	ACC (%)	AUC	ACC (%)
T1-weighted	Calf	0.892	87.0	0.892	82.6	0.796	82.6	0.837	91.3
	Thigh	0.827	80.0	0.710	72.0	0.760	80.0	0.840	84.0
	Calf+thigh	**0.953**	**92.0**	0.917	88.0	0.880	92.0	0.913	88.0
Water-only	Calf	0.846	81.6	0.904	84.2	0.890	78.9	0.872	89.5
	Thigh	0.893	88.0	0.830	76.0	0.827	84.0	0.860	76.0
	Calf+thigh	0.907	84.0	0.867	80.0	0.907	84.0	0.913	88.0
Combined	Calf	0.880	88.0	0.870	84.0	0.917	89.5	0.927	88.0
	Thigh	0.913	88.0	0.863	84.0	0.857	80.0	0.833	76.0
	Calf+thigh	0.953	88.0	0.920	92.0	0.933	92.0	0.917	89.0
Mercuri	Calf	0.847	84.0	0.903	84.0	0.793	80.0	0.840	88.0
	Thigh	0.900	84.0	0.890	88.0	0.863	84.0	0.830	80.0
	Calf+thigh	0.953	88.0	0.950	88.0	0.907	84.0	0.920	84.0

AUC, the area under the curve; ACC, accuracy; LR, logistic regression; kNN, k-Nearest Neighbors; RF, random forest; XGBoost, eXtreme Gradient Boosting. Bold indicates the highest AUC (0.953) and accuracy (92.0%) in the TM for “calf+ thigh” images.

### 3.4. Performance and clinical application of different models

**Figure 4:**
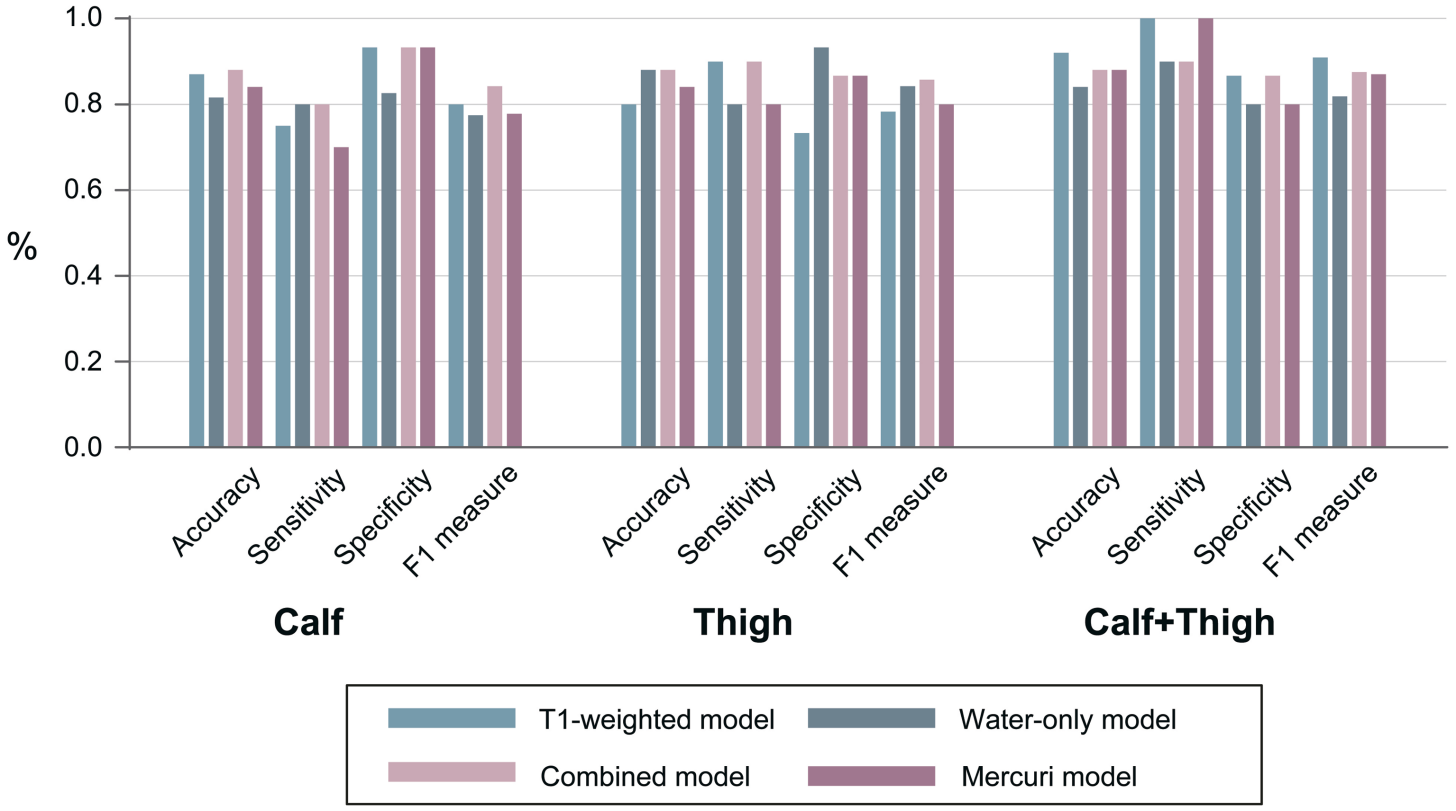
The performances of all models. The accuracy, sensitivity, specificity, and F1 measures of the prediction models were based on the calf, thigh and “calf + thigh” muscular images.

**Figure 5:**
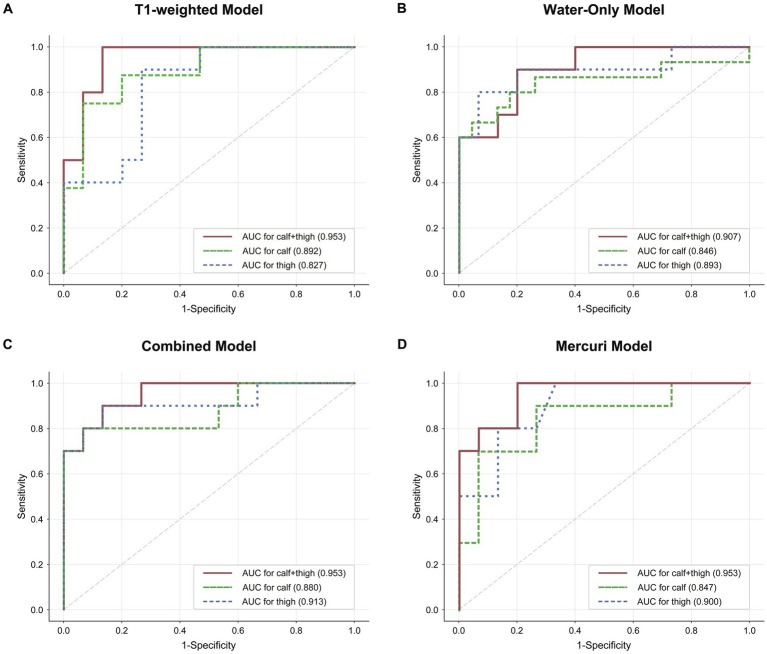
The discrimination of radiomics and the Mercuri scale model. **(A–C)** Graphs show Receiver operating characteristic (ROC) curves and area under curve (AUC) of the three radiomics models for calf, “calf + thigh” images. **(D)** The ROC and AUCs of the Mercuri model for calf, thigh and “calf + thigh” images.

A radiomics TM, WM along with CM, and the Mercuri model were built for calf, thigh and “calf + thigh” images separately. The performances of the radiomics and Mercuri models are illustrated in Figure 4. For calf images, the TM achieved a sensitivity of 75.0% with an accuracy of 87.0%, the WM 80.0% with 81.6%, the CM 80.0% with 88.0%, and the Mercuri model 70.0% with 84.0%. For thigh images, the TM achieved a sensitivity of 90.0% with an accuracy of 80.0%, the WM 80.0% with 88.0%, the CM 90.0% with 88.0%, and the Mercuri model 80.0% with 84.0%. For calf and thigh images, the TM achieved a sensitivity of 100% with an accuracy of 92.0%, the WM 90.0% with 84.0%, the CM 90.0% with 88.0%, and the Mercuri model 100% with 88.0%. Based on the ROC analysis, the performance of the radiomics models for the calf images was moderate, and the AUCs of the TM, WM, and CM were 0.892, 0.846, and 0.880. The models for thigh images showed good performance (AUC_TM_ 0.827; AUC_WM_ 0.893; AUC_CM_ 0.913). The models for the “calf + thigh” images had better performance than those for calf and thigh images (AUC_TM_ 0.953; AUC_WM_ 0.907; AUC_CM_ 0.953). The AUCs of the Mercuri model for the calf, thigh, and “calf + thigh” images were 0.847, 0.900, and 0.953 (Figure 5).

## 4. Discussion

The present study proves that radiomics analysis of MR images can be employed in calf, thigh and “calf + thigh” muscles to discriminate two myopathies, LGMDR2 and IMNM, that can be misdiagnosed in clinical practice. Based on several machine learning classification algorithms, the various MRI-based radiomics models were constructed to differentiate these two diseases, and the LR classifier was selected as the optimal machine learning model with the highest accuracy and AUC. We compared the performance of the TM, WM, and CM based on the LR model, and the models for the integration of calf and thigh muscular images showed better performance than those for calf-only or thigh-only images. Among these, TM for “calf + thigh” images presented the best value. Moreover, the AUC values of the radiomics models were higher or equal to those of the Mercuri model obtained from a visual analysis of the radiologists, revealed higher discrimination efficiency and gained great clinical net benefits, which can help clinical differential diagnosis of these two diseases. This study identifies that radiomics could be applied to muscle MRI to diagnose myopathies, even with limited cases. At present, our study is the only one that uses machine learning-based radiomics analysis of lower-limb muscle MRI to differentiate LGMDR2 and IMNM.

Muscle MRI has been developed as an essential tool for diagnosing myopathies because it can be used either for global assessment of each muscle or for compositional assessment noninvasively to assess the characteristic radiological patterns (8, 10). Traditionally, T1-weighted sequence and STIR sequence were used for muscle fatty infiltration and edema in the MRI evaluation of myopathy (29, 30). Previous studies on using the Mercuri scale based on the T1-weighted sequence to identify the different patterns of myopathies suggested that this visual scoring could be advantageous in conveniently applied in clinical practice, especially in primary care hospitals (8, 31). Some studies reported selectively involved pattern characterization of muscles evaluated by the Mercuri scale in LGMDR2 and IMNM patients (8, 9) and compared the distributions and characteristics of intramuscular fatty infiltration of lower limbs between these two diseases (5). However, different muscular disorders may have various patterns and degrees of muscular fatty infiltration, edema or atrophy (7). Therefore, the diversity of patterns and the similarities in many patterns can complicate the radiological diagnosis and cause limited inter-observer reproducibility, which could be affected by the skill of each radiologist (32). And Verdú-Díaz et al. built machine learning models using the Mercuri score to classify 10 different MDs, and the models showed almost perfect accuracy (33). Compared to this study, the main strength of radiomics technique using MRI data is that subjective visual evaluation is not required for its analysis. Although some authors believe that the radiomics approach may achieve excellent performance in the radiologic diagnosis of muscles, this method has seldom been applied to the study of myopathies (34, 35). To our knowledge, our results imply that the radiomics features seem to include more details contributing to the further diagnosis of muscular pathological changes than visual analysis.

Radiomics analysis of MR images is a noninvasive tool that can reveal the underlying pathophysio-logical changes. Several authors have confirmed the potential of radiomics analysis of muscle MRI to be used to diagnose various muscular diseases. In experimental studies of animal models with muscle disorders, radiomics analysis could be used to distinguish mouse models covering different muscular dystrophic phenotypes (36), and radiomics features enabled differentiation between the normal and the golden retriever muscular dystrophy dogs with different disease progression (37). Regarding human subjects, Lee et al. demonstrated a correlation between the radiomics features of T1-weighted images and fat fraction obtained by an MRI quantitative evaluation method, the DIXON technique. Furthermore, these features differed between Charcot–Marie-Tooth patients and controls (34). Akinci et al. reported that the radiomics models of various muscles performed better than the fat fraction (FF) models to differentiate cerebral palsy from healthy children to show that the radiomics features of muscles are more sensitive than FF (38). Nagawa et al. classified different disease subgroups in idiopathic inflammatory myopathies (IIMs) patients, including dermatomyositis, amyopathic dermatomyositis and polymyositis based on a machine learning model constructed by muscle MRI radiomics features to conclude that machine learning-based radiomics model of muscle MRI is a valuable objective tool for radiologic differential diagnosis (11).

Identifying optimal machine learning algorithms is crucial to ensure the high efficiency and reliability of radiomics models; thus, we trained four different classifiers in this study. LR performed best of all classifiers because complex models needed more training samples (39). The optimal machine learning classifiers built the TM, WM, and CM. Water-only images from the IDEAL technique rather than the conventional STIR sequence were included and extracted features in our study. Compared to the commonly performed STIR sequence, water-only images reconstructed by FSE-IDEAL allow equal or better fat suppression (40), which is more beneficial for assessing edema. Carlier and Quijano-Roy highlighted that T1-weighted images are comparable to the fat-only images obtained with the Dixon method, giving similar qualitative information (41). In addition, we used T1-weighted rather than fat-only images in our study because of a better comparison between the radiomics analysis and the visual grade system based on the Mercuri scale. Therefore, we have chosen the T1-weighted sequence and water-only sequence according to the data available to us. The radiomics features in each model were from calf, thigh and “calf + thigh” images, respectively. The difference in the distribution and severity of muscular fatty infiltration and atrophy between LGMDR2 and IMNM can be clearly shown in the T1-weighted sequence (5). Water-only images, including the water signal without fat interference, can exhibit edema of muscle and muscular fascia (18). As for the calf-only images, the accuracy of the TM, WM, and CM constructed based on LR model were 87.0, 81.6, and 88.0% in differentiating LGMDR2 from IMNM. The corresponding AUCs were 0.892, 0.846, and 0.880, respectively. As for the thigh-only images, the CM, TM, and WM accuracy was 80.0, 88.0, and 88.0% in differentiating LGMDR2 from IMNM. The corresponding AUCs were 0.827, 0.893, and 0.913, respectively. As for the integration of calf and thigh images, the accuracy of the TM, WM, and CM constructed based on LR model was 92.0, 84.0, and 88.0% in differentiating LGMDR2 from IMNM. The corresponding AUCs were 0.953, 0.907, and 0.953, respectively. In comparison, the accuracy of the Mercuri model for calf, thigh, and “calf + thigh” images were 84.0, 84, and 88%, while the corresponding AUCs were 0.847, 0.900, and 0.953. The results demonstrated that the radiomics models had higher or equal diagnostic performance, compared with Mercuri model constructed based on visual analysis, but this radiomics tool achieved more diagnosis accuracy, consistent with the study of Lee et al. (34). The results also suggested that compared with the water-only sequence, the T1-weighted sequence of calf-only or “calf + thigh” muscles had higher potential application in distinguishing these two diseases. Perhaps this is because some patients with LGMDR2 were not at the early stages of the disease, while calf muscles were involved by fatty infiltration and acute muscle edema in thigh muscles were partial relief. Yang et al. reported that patients with LGMDR2 had a longer interval of onset to first visit than patients with IMNM (5). Besides, some residual fat signal in water-only images may result in mistaking the fat signal for an edema signal (Figure 2), making it difficult to rely on muscle edema to identify the two diseases, which could explain the lower AUC of the water-only model in this study.

Moreover, this study further confirmed the requirement to analyze muscle MR images in two areas: thighs and calves. Thus, for calf-only or thigh-only images, the performance of the models is unsatisfactory. Although with a deeper understanding of LGMDR2 and IMNM recently, the result of our radiomics models and the Mercuri analysis reflects diagnostic difficulties, even for musculoskeletal imaging specialists, in distinguishing the two diseases based on MR images acquired from only the calf or only the thigh area without the help of clinical information. MR muscle images of a single area could have low accuracy, particularly in myopathies with minimal fatty infiltration or edema. In addition, the performance of the WM and CM for thigh-only images (AUC 0.893, 0.913) was better than those for calf-only images (AUC 0.846, 0.880) except the TM, and the reason might be the different patterns of muscle involvement between LGMDR2 and IMNM. A proximal lower-limb dominant involvement was reported in IMNM patients (42), while LGMDR2 patients involved at least one calf muscle (8). Also, muscle edema with the adductor magnus was evident in IMNM patients, but often absent in LGMDR2 patients (5). Thus, this result emphasized again that combined calf and thigh images to analyze myopathies could provide more diagnostic information.

Several limitations exist in this study. Firstly, the main limitation is that the sample was a small and imbalanced number and should be increased to build a more generalizable radiomics model. Also, further research is to detect whether radiomics analysis could be applied to differentiate a variety of myopathies besides LGMDR2 and IMNM. Secondly, the patients need help to stand the long image acquisition process, makes it difficult to perform whole-body MRI, which forces us to lose a large amount of muscle data from other areas, especially the body trunk and shoulders. Thirdly, considering clinical, serological, and pathological data of myopathies may have a large capacity for diagnosis, it is crucial to build a comprehensive classification model based on the combination of radiomics features and these data. Moreover, the visual score of the degree of muscle edema that might improve the efficacy of the visual model should have been considered. Fourthly, we did not screen these two myopathies according to the duration and severity of the disease. Thus, patients with advanced myopathy with marked fatty replacement or edema may affect the reproducibility of the radiomic model. To reach a practical utility, our radiomics model should be further trained with the various degrees of fatty infiltration or edema of muscle, respectively. Then, although this radiomics tool may differentiate LGMDR2 from the IMNM patients, its role in differentiating the two entities is limited. Because fatty replacement is common in many hereditary myopathies, including various LGMD subtypes. Lastly, this radiomics model did not rely on visual assessment, but it relied on subjective manual segmentation, which may be avoided by further using automatic muscle segmentation.

## 5. Conclusion

In conclusion, the radiomics analysis using the combination of calf and thigh muscle MR images can effectively differentiate LGMDR2 and IMNM, and the models constructed by the LR machine learning classifier has the highest AUC, with better performance than Mercuri visual grade system. The approach should be further applied to a large cohort of patients with a broader range of myopathies to optimize and validate this suggested model.

## Data availability statement

The raw data supporting the conclusions of this article will be made available by the authors, without undue reservation.

## Ethics statement

The studies involving humans were approved by the Institutional Review Board of Huashan Hospital, Fudan University (KY2019-409). The studies were conducted in accordance with the local legislation and institutional requirements. The ethics committee/institutional review board waived the requirement of written informed consent for participation from the participants or the participants’ legal guardians/next of kin because our study is the retrospective study, and we only used their MRI images.

## Author contributions

PW: conceptualization, investigation, and writing—original draft. HZ: data curation and writing—review and editing. QX: conceptualization, methodology, and funding acquisition. JL: methodology and visualization. SL: resources and methodology. XG: data curation. ZL: supervision, funding acquisition, and writing—review and editing. DY: conceptualization, data curation, and writing—review and editing. All authors contributed to the article and approved the submitted version.

## Footnotes

^1^ https://www.python.org/getit/
